# Photochemical Stability of a Cotton Fabric Surface Dyed with a Reactive Triphenodioxazine Dye

**DOI:** 10.3390/polym13223986

**Published:** 2021-11-18

**Authors:** Liliana Rosu, Cristian-Catalin Gavat, Dan Rosu, Cristian-Dragos Varganici, Fanica Mustata

**Affiliations:** 1 Centre of Advanced Research in Bionanoconjugates and Biopolymers, “Petru Poni” Institute of Macromolecular Chemistry, 700487 Iasi, Romania; lrosu@icmpp.ro (L.R.); fmustata@icmpp.ro (F.M.); 2 Department of Biomedical Sciences, University of Medicine and Pharmacy “Gr. T. Popa”, 700115 Iasi, Romania; ccgavat70@yahoo.com

**Keywords:** triphenodioxazines dyes, cotton fabrics, photochemical stability, color modifications

## Abstract

The paper describes the photochemical stability of a commercial triphenodioxazine dye (Reactive Blue_204) linked onto a cotton fabric. Preliminary studies have shown that as a result of irradiation, the dye and its photodegradation products can pass directly onto the skin under conditions that mimic human perspiration and cause side-effects. The cotton dyed fabric was photo irradiated at different time intervals. Standard methods were employed to evaluate the color strength at various levels of pH, temperature, dyeing contact time, and salt concentration. The influence of UV radiation at different doses (λ > 300 nm) on the structural and color modifications of the dyed cotton fabrics was studied. Structural modifications before and after irradiation were compared by applying FTIR, UV–Vis, and near infrared chemical imaging (NIR–CI) techniques. Color modifications were investigated with the CIELAB system. Color differences significantly increased with the irradiation dose. High irradiation doses caused changes in the dye structure.

## 1. Introduction

Synthetic and natural dyes have found a wide range of industrial purposes, from the well-known fabric dyeing to optical data storage, dyes for liquid crystal displays, fluorescent labeling, and light-emitting diodes. Therefore, growing research interest is focusing on enhancing dyeing efficiency and/or photochemical properties with regards to eco–friendly processes, low toxicity, and manufacturing costs [1]. Every year, over 10.000 commercially available synthetic dyes are used in the coloring stage of different industrial processes. Of these, almost 50% are reactive dyes with a global production of over 700.000 tons. The reactive dyes have the ability to form covalent bonds with the material fibers through a nucleophilic mechanism [2]. Most dyes are ecologically hazardous [3] and toxic to human health [4]. The human body comes in daily contact under any circumstance with these reactive dyes through clothing, underwear made of cotton. Long-time workers in the dye industry are exposed to serious health issues, such as different forms of dye-induced cancers, reproductive and central nervous system damage, brain and liver damage, and renal dysfunction [5,6,7]. The study of the toxic effects exerted by some of the dyes on the skin will be in the future an important milestone in their widespread use in the dyeing processes of cotton textiles. Therefore, the removal of reactive dyes from cotton textiles in humid conditions remains a challenge in addressing environmental and health issues [8].

The origin of the first triphenodioxazine reactive dye dates back to the mid-1970s [9,10]. The triphenodioxazine chromophore was designed to gradually replace the anthraquinone one [11]. In general, triphenodioxazine reactive dyes generate persistent bright and blue shades on cotton. They have gained an increased share of the blue shade area due to their very high color strength. They also imply low production costs. An example in this sense is the Reactive Blue_204 (RB_204) Color Index (C.I.) [12,13].

Of all natural fibers, cotton is amongst the most popular, due to outstanding properties, such as abrasion resistance, softness, air permeability and hygroscopicity. Due to their ability to bind to the surface of the fibers by covalent bonds, reactive dyes such as Blue_204 are often used in paint formulations for fabrics from cotton fibers. It is because of such properties, which create significant advantages, that reactive dyes are considered the best dyes for cotton to date [14,15]. Although a series of papers report studies on the photostability of reactive dyes in solution [16,17,18], the photochemical behavior of cotton dyed with Reactive Blue_204 remains a challenge. In a previous paper, the influence of UV radiation with λ > 300 nm on the photochemical stability between the cellulosic support and four reactive dyes with azo-triazine structure was studied [19].

In the present paper, the investigations toward the photochemical stability of the bond between cotton fibers and a reactive dye with triphenodioxazine structure (Reactive Blue_204) have been expanded.

## 2. Materials and Methods

### 2.1. Materials

The reactive dye RB_204 was previously purified by recrystallization from methanol (Chimopar, Bucuresti, Romania). Some additional data such as chemical structure, UV absorption peaks (λ_max_), and the molecular weights (M_w_) that characterize the reactive dye are presented in Table 1.

A fabric, manufactured mainly of alkaline cleaned and bleached cotton fibers, was obtained from a commercial source (IASITEX S.A. Iasi, Romania).

A computerized painting installation Mathis Polycolor Uniprogrammer 2002 type, manufactured by Swiss concern Werner Mathis AG (Niederhasli, Switzerland), was used. Dyeing was performed with Blue_204 reactive dye solutions, concentrations of 1, 3, and 5% aqueous solutions, using the technique of batch dyeing.

In metal capsules made of stainless steel with tight lids containing calculated amounts of reactive dye, sodium sulfate solution (concentration 50 g/L) (which had the role of electrolyte to improve the absorption and affinity of the reactive dye to the cotton substrate) and distilled water were introduced, reported to the mass of textile material (100% cotton) and the total volume of solution in the fleet. The temperature in the paint bath was raised progressively from 21 °C (room temperature) to 80 °C, with 5 °C min^−1^ heating rate and held another 10 min at this value. The heating was stopped and the calculated amounts of sodium hydroxide (400 g/L) and sodium carbonate (5 g/L) were added. The alkalization process with carbonate and sodium hydroxide followed, in order to reduce the affinity and increase the speed during the fixation of the dye. As the fixation proceeds with the release of acid, it must be buffered so as not to reduce the reactivity of the substrate and the dye. Then, the appliance was restarted, maintaining normal working parameters. The dyeing time was 30 min. A thermostatic bath temperature at 80 °C was kept. Finally, the paint fleet was gradually cooled, by three degrees per minute, to a temperature of 38 °C. For the entire duration of the dyeing, the metal enclosure of the device was sealed for about 60 min. After cooling, the metal capsules with the paint fleets were removed from the installation. The dyed samples were washed repeatedly with warm water and cold water and finally rinsed for 15–20 min with 60–70 mL of distilled water, at 80 °C, to completely remove the dye not fixed on the textile fabric [20].

### 2.2. Methods

#### 2.2.1. Color Modifications Measurements

The color analyses were performed with a Lovibond LC 100 manufactured by Tintometer Ltd., Amesbury, UK. A super white sulphate barium pellet was used to calibrate the device. Measurements were conducted in reflectance mode using D65-daylight illuminant at 10^0^ standard observers. The results were presented in accordance with CIE 1986. The color changes were evaluated by monitoring the variation of the lightness factor (L*) and of chromatic coordinates: redness ↔ greening (a*) and yellowness ↔ blueness (b*) before and after irradiation. In the CIELAB system, the L* parameter can have values between 0 (black) and 100 (white) and the intermediate values indicate different shades of gray. The parameter a* can have negative values (−a*), characterizing the green color, or positive (+a*) values, indicating the color red. By analogy, the negative parameter b* (−b*) indicates the color blue and when it has positive values (+b*), indicates the color yellow.

The global color variation caused by irradiation (ΔE_ab_) can be calculated using Equation (1):

(1) $$\Delta Ε*=\sqrt{{\left(L_{2}^{*}-L_{1}^{*}\right)}^{2}+{\left(a_{2}^{*}-a_{1}^{*}\right)}^{2}+{\left(b_{2}^{*}-b_{1}^{*}\right)}^{2}}$$

In Equation (1), the 1 and 2 indexes correspond to the parameters recorded before and after irradiation.

#### 2.2.2. Ultraviolet-Visible (UV-Vis) Absorption Spectra

The UV-Vis spectra were recorded with a SPECORD 200 spectrometer (Analytik, Jena, Germany). The quantitative determination of the dyes detached from the cellulosic fibers, as a result of exposure of fabrics to UV radiation, were made with UV-Vis Cole Palmer 1100 RS apparatus (Antylia Scientific, Vernon Hills, IL, USA) provided with Unico 1100 SS-1.11 version software (Unico Inc., Caledonia, WI, USA). Measurements were carried out at the visible wavelengths corresponding to the absorption peaks that characterize each dye. Concentrations of the extracted dyes were calculated using the standard previously plotted curves. The dyes extraction solutions were prepared according to the standard SREN ISO 105-E04, January 1998, classification index L22, Textiles. The tests for colour fastness at perspiration were undertaken using the prescriptions given below [21]. For the alkaline extraction, a freshly prepared solution containing 0.1 g α-L alanine (Sigma Aldrich, Berlin, Germany), 1 g NaCl (Chemical Company, Iasi, Romania) and 0.5 g Na_2_HPO_4_·2H_2_O (Sigma Aldrich, Berlin, Germany) was used. The solid substances were dissolved in 200 mL distilled water and the pH values were adjusted to 8 using a 0.1 mol L^−1^ NaOH (Chemical Company, Iasi, Romania) solution. Similarly to the alkaline extraction, a freshly acid solution containing 0.1 g α-L alanine, 1 g NaCl, and 0.5 g NaH_2_PO_4_·2H_2_O was prepared. The solid substances were dissolved in 200 mL distilled water and the pH values were adjusted to 5.5 using a 0.1 mol L^−1^ NaOH solution. The pH value was monitored with an AB 15 Plus Cole Parmer device provided with a glass electrode Accumet BASIC.

#### 2.2.3. Irradiation

Cotton fabric samples with 2 cm × 2 cm (width × length) surface dyed with concentrations of reactive dye (1%, 3% and 5%) were irradiated at 25, 50, 75, and 100 h, with a medium pressure OSRAM HQE-40 lamp, as an artificial light source, in the range 240–400 nm and of 100 W power. Irradiation was undertaken in air with the aid of a rotating hexagonal prism shaped device, used as sample carrier and having the light source put on the prism’s central ax. During irradiation, samples were protected against thermal degradation by a distilled water filter and a fan. A quartz/borosilicate filter with a maximum transparency at 365 nm was used to remove the more energetic radiations (λ < 300 nm), not present in the natural solar light spectrum. The irradiance value, measured at a distance of 60 mm from the source, was 9.7 mW cm^−2^. This value was about 4.4 times higher than the average UV irradiance measured outdoors on a clear summer day (2.2 mW cm^−2^). A PMA 2100 radiometer provided with a UVA detector, type PMA 2110 (Solar Light Co., Glenside, PA, USA) was used for measurements of the irradiance value and radiant exposure, which is dependent on the irradiation time [22]. The temperature measured inside of the irradiation device was 20–22 °C and the relative air humidity (RH) was 53%. A thermo-hygrometer model JK-HTM-3 (Shanghai Jingke Scientific Instrument Co., Shanghai, China) was used to monitor the temperature and RH values during irradiation.

#### 2.2.4. Near Infrared Chemical Imaging Spectroscopy (NIR-CI)

Near infrared chemical imaging (NIR-CI) spectra were recorded on a Specim’s Ltd. (Oulu, Finland). Sisu CHEMA device controlled with Evince software package for processing the original image data. The system includes a chemical imaging workstation for 1000–2500 nm NIR 130 domains. The original image for each sample was taken with a NIR model spectral camera, respectively, an imaging spectrograph type ImSpector N17E with 320 and 640 pixel spatial resolution at a rate of 60–350 Hz.

#### 2.2.5. Fourier-Transform Infrared Spectroscopy (FTIR)

FTIR spectra of the dyed samples were acquired with a Vertex 70 spectrophotometer (Bruker, Karlsruhe, Germany) equipped with an ATR crystal plate (MIRacle^TM^, New Kowloon, Hong Kong, China) made of diamond in the range 4000–400 cm^−1^ and processed with OPUS 6.5 software. The spectra were recorded at a resolution of 4 cm^−1^ by 16 scans.

## 3. Results

### 3.1. Color Modification Studies

The color characteristics, calculated with Equation (1), for the fabric dyed with RB_204 at three different concentrations are presented as function of irradiation time and dose in Table 2.

**Figure 1:**
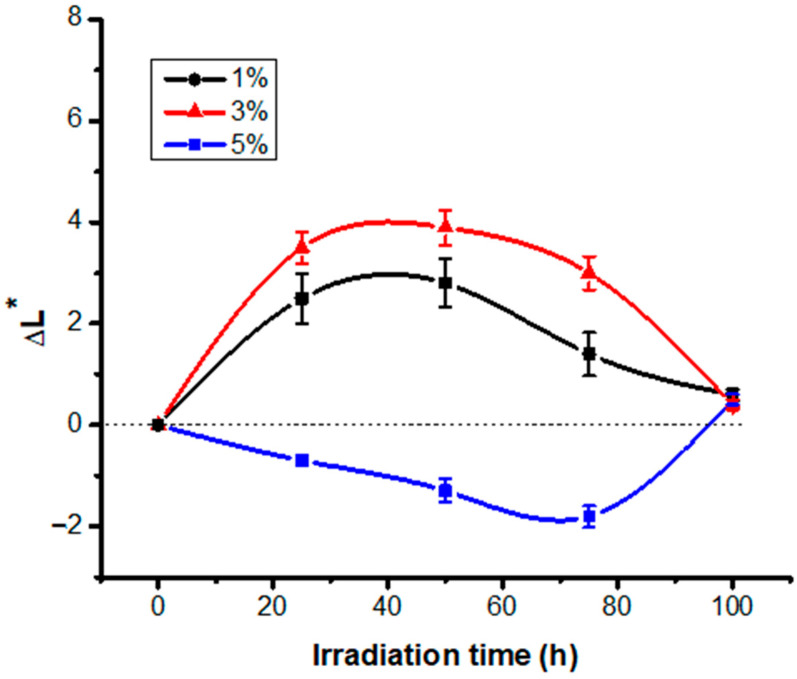
ΔL* variation recorded for RB_204 samples during irradiation.

From Table 2, it can be seen the darkening of the dyed fabric samples as the concentration of dye solution increases [19]. For additional data, the variation of the other color parameters was monitored depending on the exposure time. Thus, the increase of L* values in the first 50 h of irradiation was observed, especially for the samples dyed with solutions containing 1% and 3% RB_204 dye. UV radiation acts quickly and visibly when the dye layer is thin and so it photo-degrades easily and quickly. For these samples, a bleaching tendency of the dyed fabrics in the first 50 h of exposure may be observed, which confirms the reduction in the amount of RB_204 fixed on the fiber as a result of photo-degradation. The subsequent decrease of the L* factor when irradiation has been prolonged up to 100 h can be explained by the photo-degradative action of the UV radiation on the cotton support impoverished in dye, which causes a slight darkening. The fabric dyed with a more concentrated dye solution (5%) seems more resistant to photo-degradation. In this case, the L* values decrease slightly from 19.7 to 17.9 up to 75 h of irradiation after which by continuing the exposure, there is an increase of lightness factor over the value of the unexposed sample. The decrease of the L* values may be a result of some photo-degradation products accumulation that resulted from the RB_204 dye, fixed in superior quantities compared with the previous samples due to the higher concentration of the solution used for dyeing. In this case, the dyed fabric faded only after 100 h, as a consequence of a longer time needed for the textile support to lose enough dye to reduce the protection. These observations are supported by the variation of the ΔL* difference with the irradiation time described in Figure 1. The dye and textile support have totally different chemical structures. That is why the two substances degrade through separate mechanisms and at different rates. Even the intensity of the UV radiation felt on the surface of the two materials is different, the dye being more exposed compared to the textile support at least in the first 50 h. Finally, the structural changes of the fabric dyed with RB-204 after irradiation occur both in the dye and in the textile support following different mechanisms. It seems that in the dye, the destruction of chromophores takes place having as a consequence its discoloration and the cellulosic support undergoes photo-oxidation reactions accompanied by depolymerization, supported by the tendency of yellowing of the material after irradiation (b* values increase).

The values of the parameter a* gradually decrease with the irradiation time regardless of the concentration of the RB_204 solution used for dyeing, as it may be seen in Table 2.

**Figure 2:**
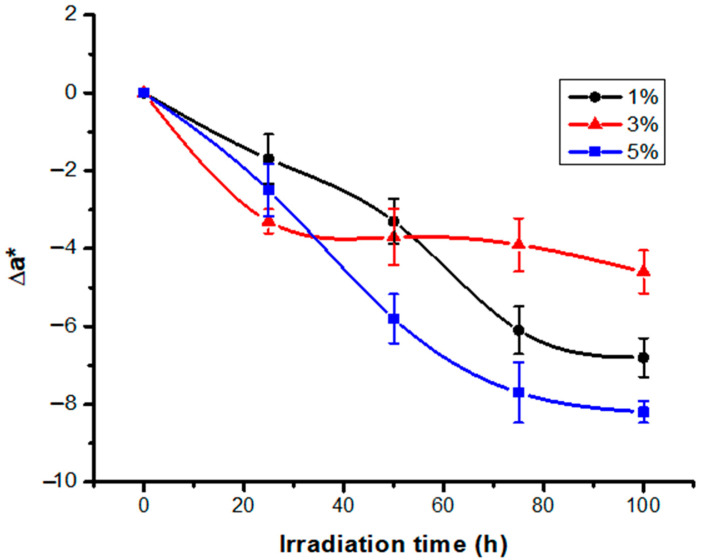
Variation of difference Δa* with irradiation time for RB_204.

The negative values of the Δa* differences presented both in Table 2 and in Figure 2 confirm that during irradiation, the dyed fabric loses its red component.

**Figure 3:**
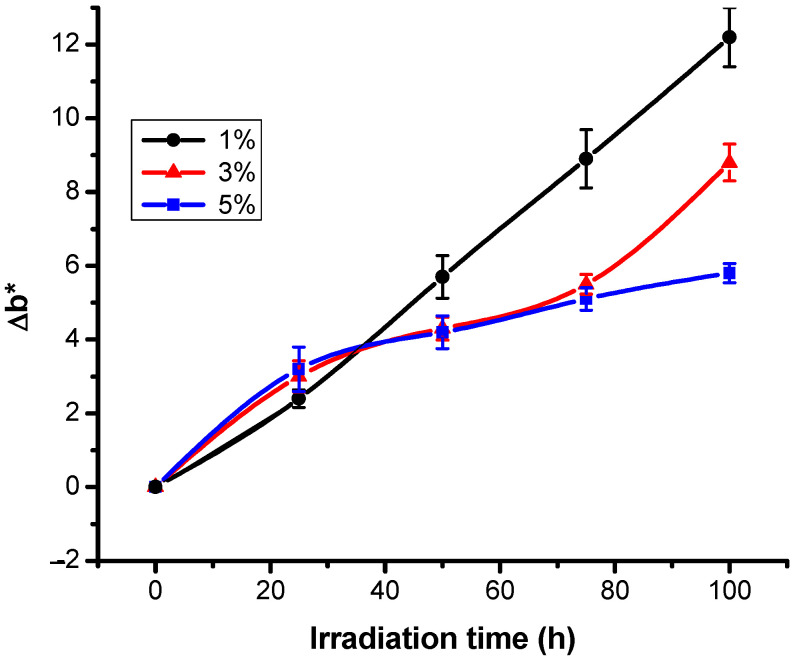
Variation of chromatic coefficients Δb* with irradiation time for RB_204.

The values of the chromatic parameter b* are negative, indicating the intense blue coloration (Table 2). It is interesting that the b* values that characterize the non-irradiated samples are between −33.3 and −34.3 and appear to be independent of the initial dye concentration. The values in the table indicate a tendency to increase the parameter b* during exposure of colored samples to UV radiation. The positive values of the Δb* differences calculated during the UV exposure of the dyed fabrics underline this trend (Figure 3). The conclusion is that UV exposure causes an increase in chromatics in the sense that the colored fabric studied on the –b* ↔ +b* axis became less blue after irradiation. If at low dye concentrations (up to 3%) there is a gradual increase of parameter b*, at 5% dye concentration, the change of b* value is steeper.

**Figure 4:**
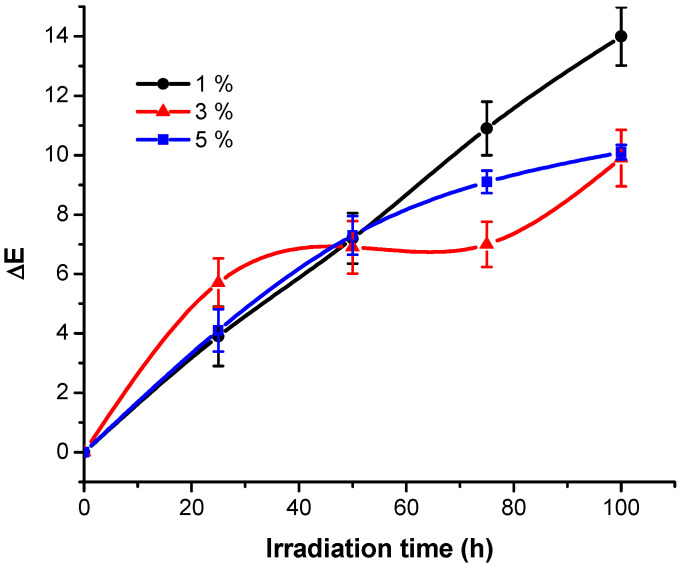
Total colour changes generated by exposure RB_204 samples under UV light.

From Table 2, one may see the influence of irradiation time and radiant exposure on the ΔE values. Thus, it can be observed that the increase of ΔE values varies proportionally with the irradiation parameters, regardless of the concentration of the dye solution used in the dyeing process (Figure 4).

This observation leads to the idea that the dye layer bounded to the cellulosic fiber is significantly affected by UV exposure. It seems that the most affected are the samples dyed with the 1% dye solution when the highest color differences were observed (ΔE = 14.0 after 100 h irradiation time). For fabrics dyed with solutions containing 3% and 5% dye, the color differences recorded during exposure are comparable (ΔE = 9.9 for 3.5% RB_204 concentration solution and 10.1 for 5% concentration solution RB_204). Figure 4 shows a general tendency to increase the ΔE values with the irradiation time. The concentration of dye seems to influence the profile of the curve evolution. There is a tendency to continuously increase the ΔE values during irradiation, regardless of the dye concentration and the values of ΔE > 10, recorded after 100 h of UV exposure, indicate very important color modifications of the dyed fabrics as a result of UV exposure.

### 3.2. Study of the Variation of Color Intensity as a Function of Irradiation Time and Different Dye Concentrations

The color intensity at the surface of the dyed fabric was evaluated before and after irradiation of samples using the spectral reflectance *F*(*r_λ_*), defined according to the Kubelka-Munk Equation (2) by the ratio between light absorption (*K*) and light diffusion (*S*) coefficients.

(2) $$F\left(r_{\lambda }\right)=\frac{K}{S}=\frac{{\left(1-r_{\lambda }\right)}^{2}}{2r_{\lambda }}$$

In Equation (2), the *r_λ_* is the ratio between the reflectance of the analyzed sample, and the reflectance measured on a Whatman paper no. 42 with known porosity [23].

**Figure 5:**
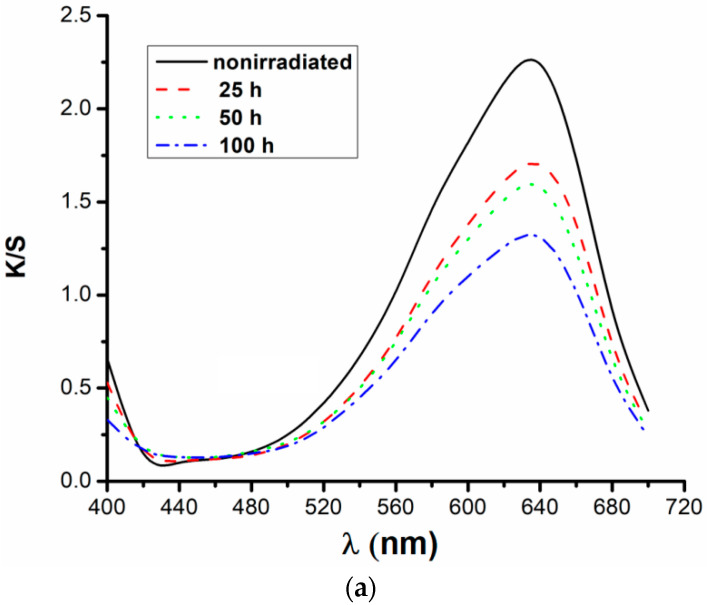

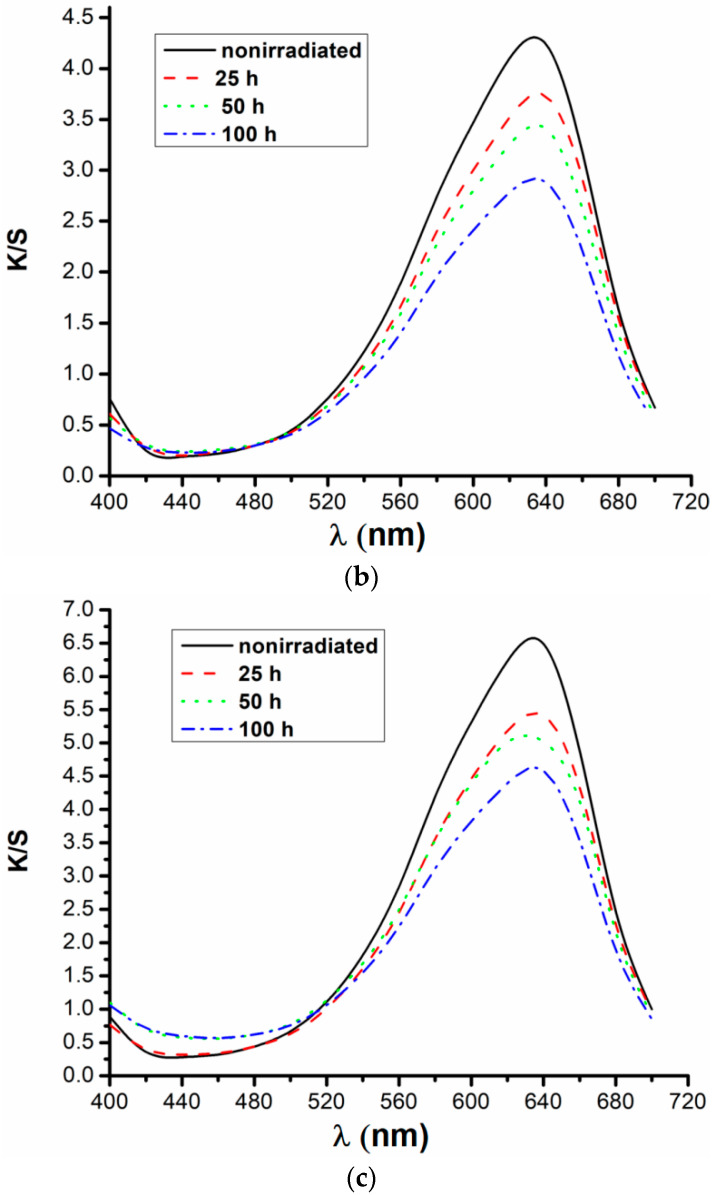
Color intensity for samples dyed with RB_204 and irradiated: (**a**) 1%, (**b**) 3%, (**c**) 5% dye.

The transformations proposed in Equation (2) allow the interpretation of maxima that appeared in the reflectance spectra as those from classic UV-Vis spectra. A graphical plotting of *K*/*S* ratio variation as a function of *λ*, for the entire visible range and dye concentration, is presented in Figure 5.

It can be observed that all the analyzed samples show wide absorptions in the wavelength range 500–700 nm with maxima at λ = 636 nm. All maxima decreased in intensity with the duration of irradiation suggesting a hypochrome shift. It is a proof that the fabric loses dye during UV exposure or the dye was photodegraded on the fiber surface. The values of color intensity (*K*/*S*), deduced from the wavelength at which the absorbance is maximum, are given in Table 3.

The degree of discoloration of dyed samples after irradiation measured at different time intervals was calculated with the following Equation (3):

% discoloration = 100 × [(*K*/*S*)_nonirradiated_ − (*K*/*S*)_irradiated_]/(*K*/*S*)_nonirradiated_ (3)

This determination aimed to highlight the degree of dye degradation on the dyed and irradiated samples (25, 50, and 100 h), compared to the non-irradiated ones, as a control.

From Table 3, it was observed that the color intensity decreases with increasing of exposure time, which indicates an increase in dye degradation on the fiber after irradiation. Based on the values obtained for the *K*/*S*, the quantities of dye existing on the textile support after irradiation were calculated.

From the analysis of Table 4, it was found that the stability of the reactive dye RB_204, expressed by the amounts of dyes remaining on the fibers, decreases with the increase of the irradiation time. Regarding the influence of the dye concentration on degradation, it was observed that the highest degradation (lowest dye stability) occurred in the case of samples dyed with a low dye concentration (1%). Following UV irradiation on the cotton sample with 1% dye, a minimum amount of reactive dye remains, compared to the samples dyed with 3% and 5% dye solutions. It was also observed that the highest color stability, materialized by the largest amounts of reactive dye remaining on the fiber after irradiation, was exhibited by the samples dyed with a high concentration of dye (5%).

### 3.3. Quantitative Spectrophotometric Analysis in UV-Vis for the Reactive Dye RB_204

**Figure 6:**
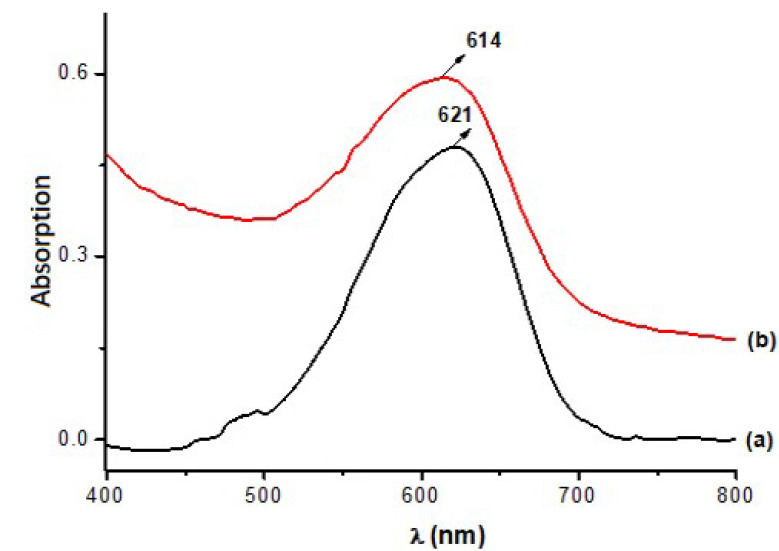
UV-Vis absorption spectra of RB_204 dye: (**a**) non-irradiated; (**b**) irradiated 100 h.

Figure 6 shows that the RB_204 dye has the maximum absorption in the visible range at 621 nm for solutions of concentration c = 8 μg/mL = 0.008 g ‰, at neutral pH.

The reduction of absorption (hypochromic effect) and the bathocrom displacement of the peak towards higher wavelength values (from 614 to 621 nm) was observed. Hypochromic modification of the UV spectrum after irradiation may be a consequence of partial destruction of the dye structure action while bathochrome modification supports the extension of the conjugate double bonds system. Hypochromic modification of the UV spectrum after irradiation may be a consequence of partial destruction of the dye structure action, while bathochrome modification supports the extension of the conjugate double bonds system. Literature reports correlate the darkening of dyes with complex photo–oxidation phenomena, leading to the formation of cromophores [19,24]. The decrease in L* values may be correlated with amine entities number and substitution degree also explaining the slight bathochrome deplacement effect and green and yellow cromophore accumulation. The hypochrome effect is generated by aromatic ring disruption [19,25].

### 3.4. Structural Modification Studies by FTIR

**Figure 7:**
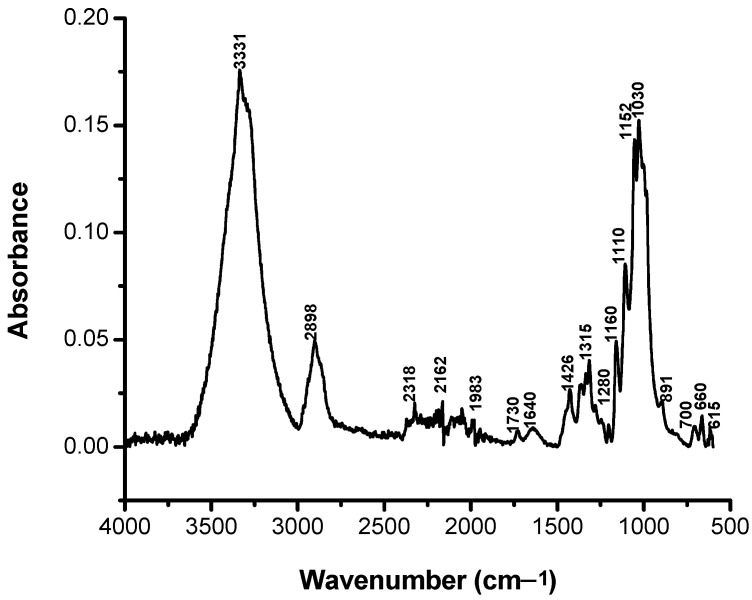
FTIR spectrum of the cotton fabric.

**Figure 8:**
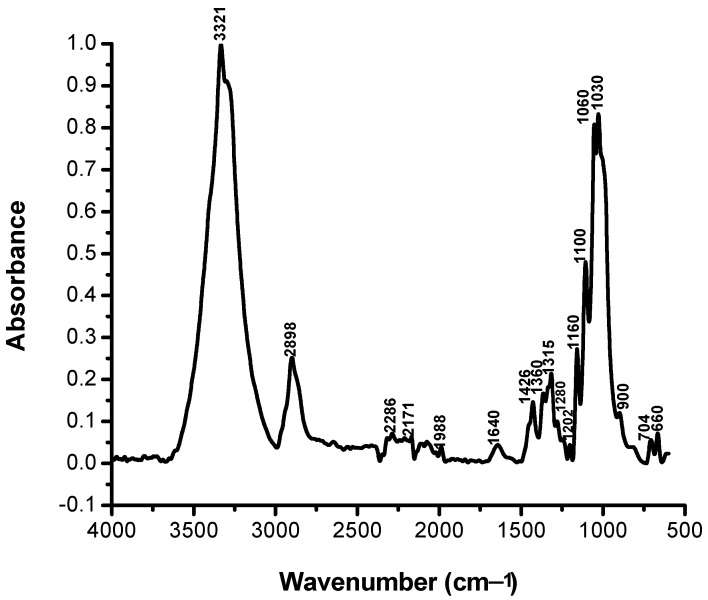
FTIR spectrum of the cotton fabric dyed with RB_204.

**Figure 9:**
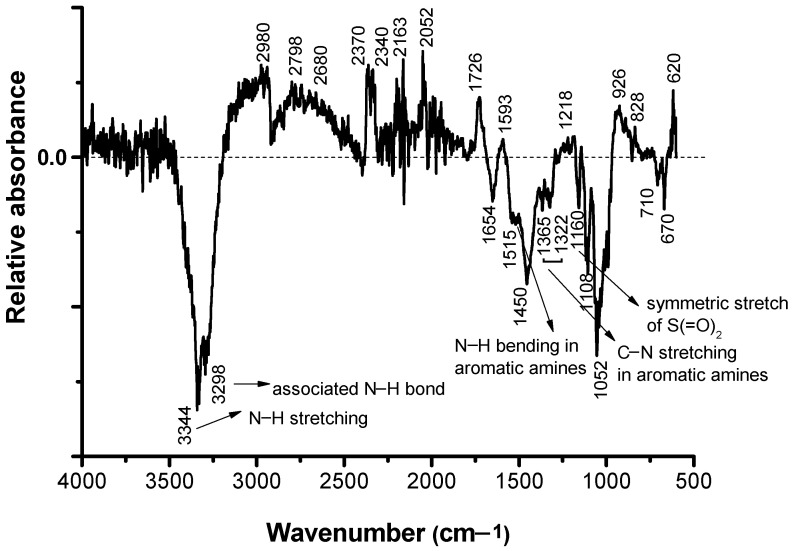
FTIR difference spectrum of the initial non-dyed cotton fabric (positive signals-upwards) and the cotton fabric dyed with RB_204 (negative signals-downwards).

**Figure 10:**
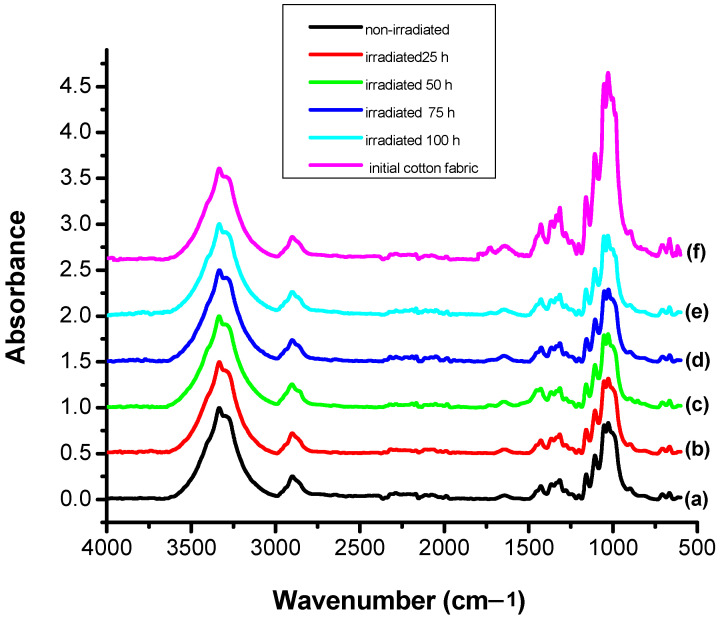
FTIR spectra of: (**a**) initial RB_204 dyed cotton fabric; (**b**) RB_204 dyed cotton fabric irradiated at 25 h; at (**c**) 50 h; at (**d**) 75 h, and at (**e**) 100 h; (**f**) cotton fabric.

**Figure 11:**
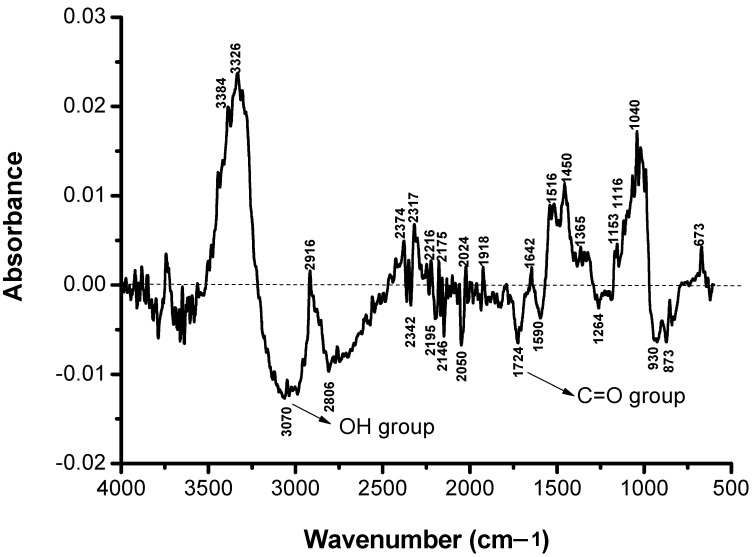
FTIR difference spectra RB_204 dyed cotton fabric non-irradiated and irradiated 100 h.

In order to characterize as rigorously as possible the loss of color intensity of the samples subjected to UV exposure based on structural changes, the FTIR spectra were recorded for the non-dyed and dyed samples before and after irradiation at different time intervals: 0, 25, 50, 75, and 100 h (Figure 7, Figure 8, Figure 9, Figure 10 and Figure 11).

It can be seen that the difference spectrum between the spectrum of the fabric and the dyed fabric (Figure 9) shows positive and negative signals. The positive signals indicate the presence of non-dyed fabric structural entities. Negative signals are new structural entities, specific to the structure of the dye on the fabric. Comparing the FTIR spectra in Figure 7 and Figure 9, corresponding to the fabric, it can be seen that the bands specific to the fabric groups decreased in intensity after dyeing. The dye-specific bands (negative area of the spectrum in Figure 9) show: valence vibrations of the secondary N–H groups (3344 and 3298 cm^−1^); stretching vibrations specific to the secondary N–H groups (1515 cm^−1^) and of the aromatic C–N bonds (1365 and 1322 cm^−1^). Also, the difference FTIR spectrum highlights the presence of bands specific to S(=O)_2_ (1160 cm^−1^) sulfonate groups [26]. The absence of the absorption band specific to the C–F connection (1344 cm^−1^) indicates the attachment of the dye to the fabric surface by covalent bonding [27]. After 100 h of irradiation (Figure 11), there is an increase in the intensity of positive signals from the difference spectrum, specific to the fabric, and a decrease in negative signals, specific to the dye, as a result of photochemical degradation of the dye. The signals specific to secondary amines (3344, 3298, 1515, 1365, and 1322 cm^−1^), as well as the absorption band characteristic to sulfonate groups of type S(=O)_2_ (1160 cm^−1^) disappear. This confirms the photodegradation of the dye or its detachment from the fabric.

Figure 7 shows the FTIR spectrum of the control cotton fabric. The appearance of a shoulder centered at approximately 1730 cm^−1^ due to the carboxyl and/or carbonyl groups is observed [28].

It is interesting to note the intensity of such a shoulder depends on the time of UV irradiation exposure. For a most thorough characterization of dye color intensity loss based on structural modifications during UV irradiation, FTIR spectra (Figure 10) were recorded for the non–irradiated cotton fabric and the dyed cotton fabric irradiated at the selected times. Three domains of interest were chosen: 2500–1900 cm^−1^, 1800–1500 cm^−1^, and 1400–1000 cm^−1^. An important problem was to identify functional groups in the structure of the dye fixed on the cotton sample, non-irradiated and irradiated for 100 h, in order to explain the transformations that take place in the dye. Different group signals within these ranges were studied in detail. A prioritary aspect in the study, based on FTIR spectroscopy, is the dye–celullose fiber ether covalent bond (Ar–O–Cell), whose stability was studied after UV irradiation in comparison with the non–irradiated sample. By comparing the FTIR spectra in Figure 10, one may observe a series of characteristic peaks in the irradiated dyed cotton spectrum within the ranges 1800–1500 cm^−1^ and 1400–1000 cm^−1^. In the range 1800–1500 cm^−1^ the non–irradiated sample exhibits three signals, of which one very pronounced with a peak at 1646 cm^−1^ between 1640 and 1651 cm^−1^ attributed to the modification of the Ar–C = C– bond after UV irradiation.

The dyed irradiated sample exposed 100 h showed more and smaller signals of which only the one in the range 1649–1650 cm^−1^ being in common with the non–irradiated sample spectrum. The characteristic absorbance frequencies of the most important groups in RB_204 dye are given in Table 5.

For a more precise identification of signals emitted by specific different chemical entities and existing bonds within the dye structure, the wavenumber range of interest between 1450 and 1850 cm^−1^ was used. In the case of the irradiated dyed cotton, there appears a signal at 1044 cm^−1^ (between 1025 and 1064 cm^−1^) and two signals (shoulders) at 1252 and 1273 cm^−1^. A third signal at 1337 cm^−1^ was identified only in the FTIR spectrum of the non-irradiated dyed sample.

The dye–fiber presents two signals specific to asymmetrical and symmetrical etheric bond vibrations υC–O–C at 1240 and 1032 cm^−1^, respectively. After 100 h of UV irradiation, it was observed that the signal from 1032 cm^−1^ disappeared and was replaced by the signals at 1025 and 1044 cm^−1^. The asymmetric vibration corresponding to the frequency at 1240 cm^−1^ has significantly decreased in profile and amplitude.

During 100 h irradiation time, modification and scission of the dye–fiber ether bond occurred. Some signals were observed at 2235 cm^−1^ for the non–irradiated dyed sample, which shifted to 2232 cm^−1^ and was attributed to some –CH– alkyl groups. Other signals of the non–irradiated dyed sample, at 2080, 2052, and 2023 cm^−1^, characterizing conjugated double bond systems from condensed aromatic entities, which underwent structural modification, completely disappeared after irradiation. The process of UV irradiation of colored cotton results in the production of free radicals and the initiation of chemical reactions, such as depolymerization, dehydrogenation, dehydroxylation, dehydromethylation, and evolvement of carbon dioxide [29,30,31,32]. Remarkable is the decrease in the intensity of the absorption band around 1730 cm^−1^, attributed to the carbonyl group, observed in the FTIR spectrum of the photoirradiated sample, which suggests that photodegradation occurs on the colored cotton sample exposed under a UV light source mainly by the chain split.

Triazine rings, mono-, di-, or trisubstituted with an alkyl or aryl carbon directly attached to the ring have at least one strong band in the region 1580–1525 cm^−1^, which is attributed to the double bond, and at least one weak band in the region 860–775 cm^−1^. It does not seem to be possible to distinguish mono-, di-, or trisubstitution, except possibly for ring CH stretch absorption for mono or disubstitution, which can sometimes be seen in the 3100–3000 cm^−1^ region. There is usually at least one band in the 1450–1350 cm^−1^ region [33].

### 3.5. Spectrophotometric Determination of the Concentration and Amount of Extracted Dye

#### 3.5.1. Results Obtained on Non-Irradiated Samples in a Neutral Medium

The absorbances of the extraction solutions, obtained in a neutral medium, using distilled water, were determined spectrophotometrically. For this purpose, non-irradiated samples were used. From the equation of the calibration line, the extracted dye concentration (μg/mL) was calculated, as well as the amount of dye extracted from the fiber as a function of the sample mass and the variation of the dye concentration as a function of extraction times was plotted.

Table 6 shows the absorbance values determined spectrophotometrically depending on the extraction times, the concentration of the extracted dye (μg/mL) and the mass of the weighed sample. The amount (μg/g sample) of RB_204 dye extracted from the fiber was also calculated, based on the volume of the extraction solution (25 mL) and the mass of the weighed sample.

**Figure 12:**
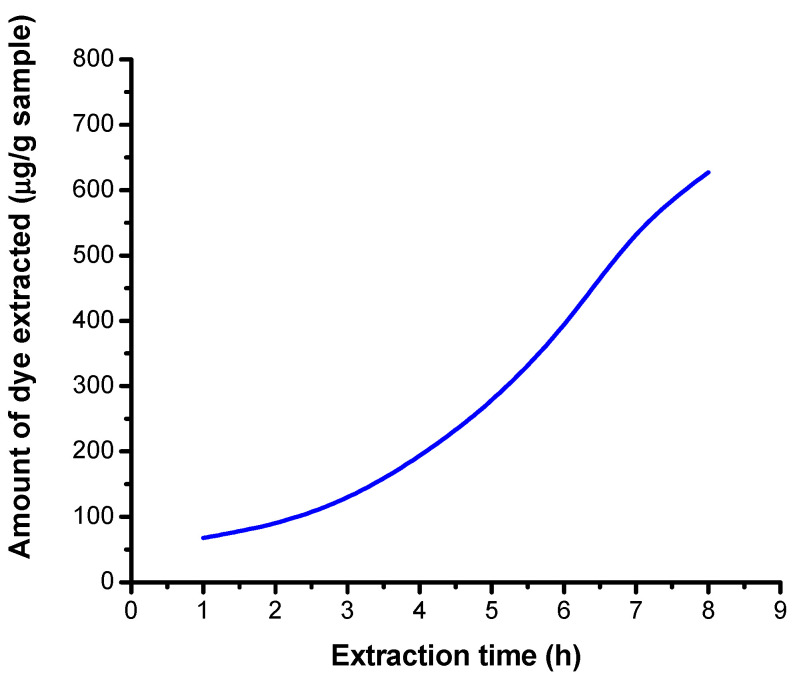
Dependence of the extracted 5% dye concentration on the extraction time in a neutral environment.

The variation of the 5% dye concentration extracted from the fiber (μg/g fiber) depending on the extraction time is shown in Figure 12.

There is an increase in the concentration of the dye RB_204 extracted from the sample irradiated with increasing extraction length.

#### 3.5.2. Results Obtained on Irradiated Samples by Extraction in Alkaline Medium

The absorbances of the solutions obtained after extraction in an alkaline medium were determined spectrophotometrically. From the equation of the calibration line, the dye concentrations extracted from the UV dyed and irradiated samples were calculated at time intervals of 1–8 h. From the concentration values were calculated the quantities of extracted dyes, relative to the mass of the cotton sample. The results are presented in Table 7.

**Figure 13:**
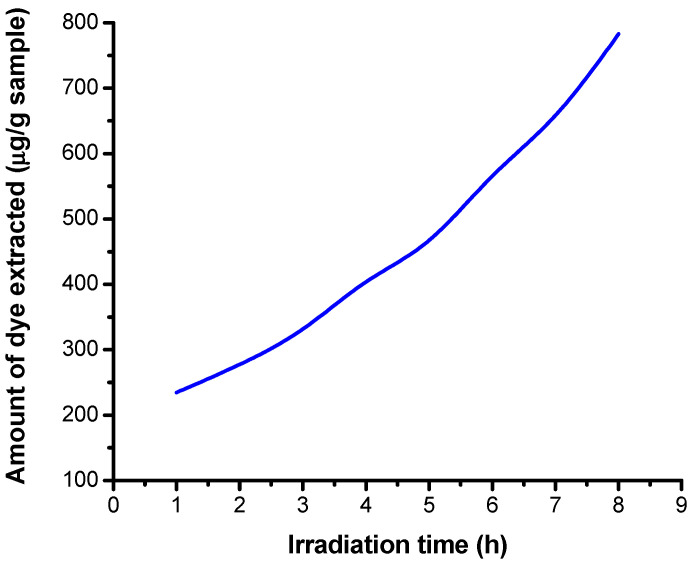
Dependence of the extracted 5% dye concentration on the irradiation time in an alkaline medium.

The variation of the amount of dye extracted in basic medium from the fiber, depending on the irradiation time is shown in Figure 13.

From Figure 13, it was observed that there is an exponential increase, in basic medium, of the amount of dye extracted depending on the irradiation time.

#### 3.5.3. Results Obtained on Irradiated Samples by Extraction in Acid Medium

The absorbances of the extraction solutions were determined using the freshly prepared acid extraction solution as a control. Then, from the equation of the calibration line, the concentration of dye extracted according to the masses of the samples (μg col./g fiber) was calculated. A total volume of 15 mL of solution was used. The variation of the concentration of dye extracted from the dyed cotton, as a function of the irradiation time, was plotted. The results are presented in Table 8.

**Figure 14:**
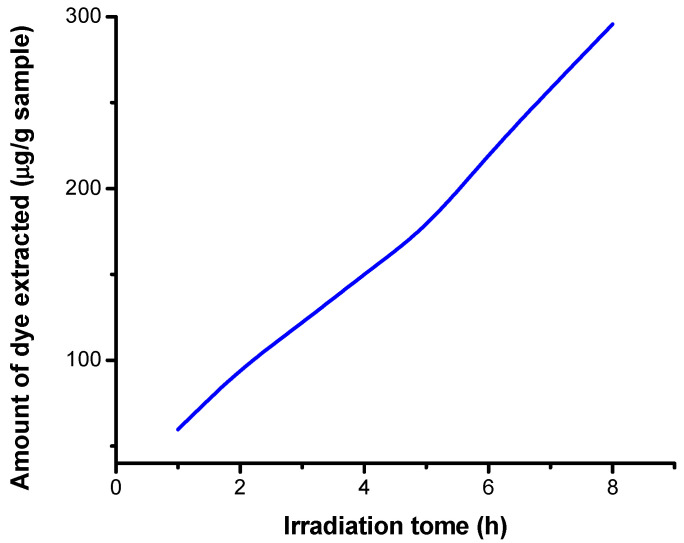
Dependence of the extracted 5% dye concentration on the irradiation time in an acid medium.

Following the analysis of the variation of the concentration of the extracted dye according to the irradiation duration (Figure 14), the increase in the concentration of the extracted dye in acid medium as a function of the irradiation duration was found. The amount of dye extracted from the dyed cotton increases with irradiation time.

The seperated dye amount from the cotton fabric is dependent on irradiation time and dose. Figure 12, Figure 13 and Figure 14 show the variation of dye ammount extracted with distilled water and low basic and acidic solutions as a function of irradiation time.

From Figure 12, Figure 13 and Figure 14, one may observe an increase in extracted dye quantity from the cellulose substrates with pH and irradiation time. This may be correlated with a more intense photodegradation process of the cellulose substrate.

### 3.6. Structural Modifications Studies by NIR-CI Spectroscopy

**Figure 15:**
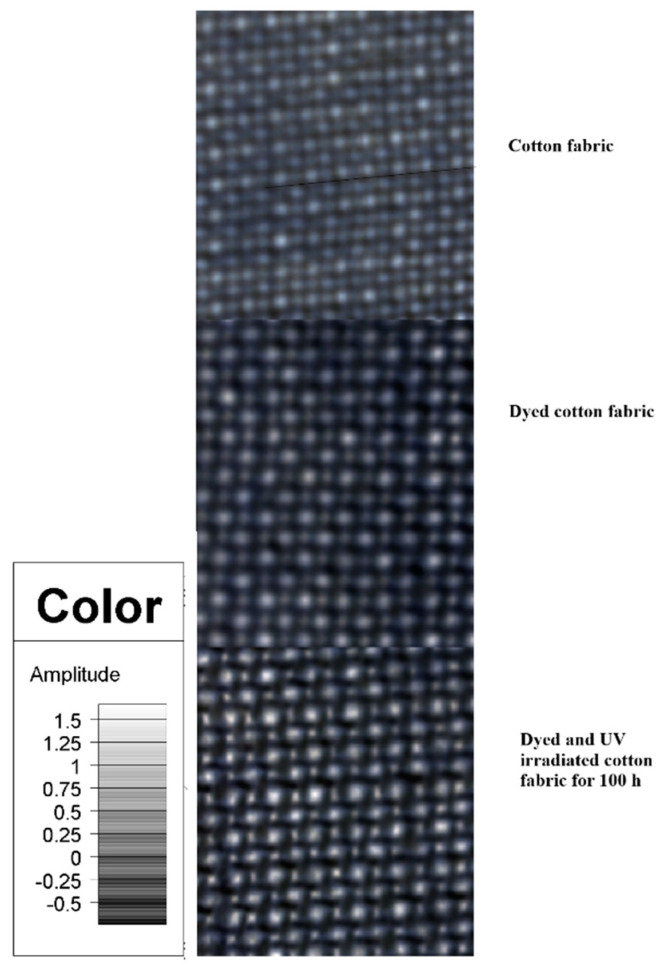
PLS-DA model for non dyed cotton fabric, dyed cotton fabric, and dyed and UV irradiated cotton fabric for 100 h.

Reactive dyes with triphenodioxazine structure are suspected of carcinogenicity. NIR-CI spectrophotometric analysis of reactive dyes with triphenodioxazine structure could be a solution to this problem. Samples of the non–dyed, dyed, and dyed and 100 h UV irradiated cotton fabrics were characterized with the aid of the NIR-CI technique. This technique is a facile and rapid analytical method for quantitative assessment and requires no special sample preparation. Device calibrations are the only time-consuming procedures. The NIR-CI method provides useful insights on sample components spatial distribution by a chemical image, enabling a sample chemical and/or physical heterogeneity degree evaluation. [34,35,36,37]. The obtained chemical images are visualized in the forms of three-dimensional blocks of data, further implied in partial least squares–dynamic analysis (PLS–DA) with the Evince software. The PLS–DA method allows the recorded spectral data to be decomposed into a small number set of classification scores. The scores are generated by correlating spectral information with response variables. Since NIR-CI offers the possibility of monitoring different functional groups modifications at the dye–cotton substrate interface [38,39]; the method was also used for monitoring chemical structures. Figure 15 shows the PLS–DA model for non–dyed, dyed, and dyed and 100 h UV irradiated cotton fabric from which one may observe the predominance of grey color in mostly all score regions. This is an indication of a good dye dispersion within the cotton textile surface.

With the aid of IR reference spectra within the NIST spectral library database, identification of each functional group experimental signals was possible and the following absorption bands values were assigned: wide band 1470–1535 nm (N–H stretch first overtone); 1860 nm (C–Cl stretch sixth overtone); 2060 nm (N–H bend second overtone or N–H bend/N–H stretch combination); 2200 nm (C–H stretch); and 2300 nm (C–H bend second overtone) (Figure 15).

**Figure 16:**
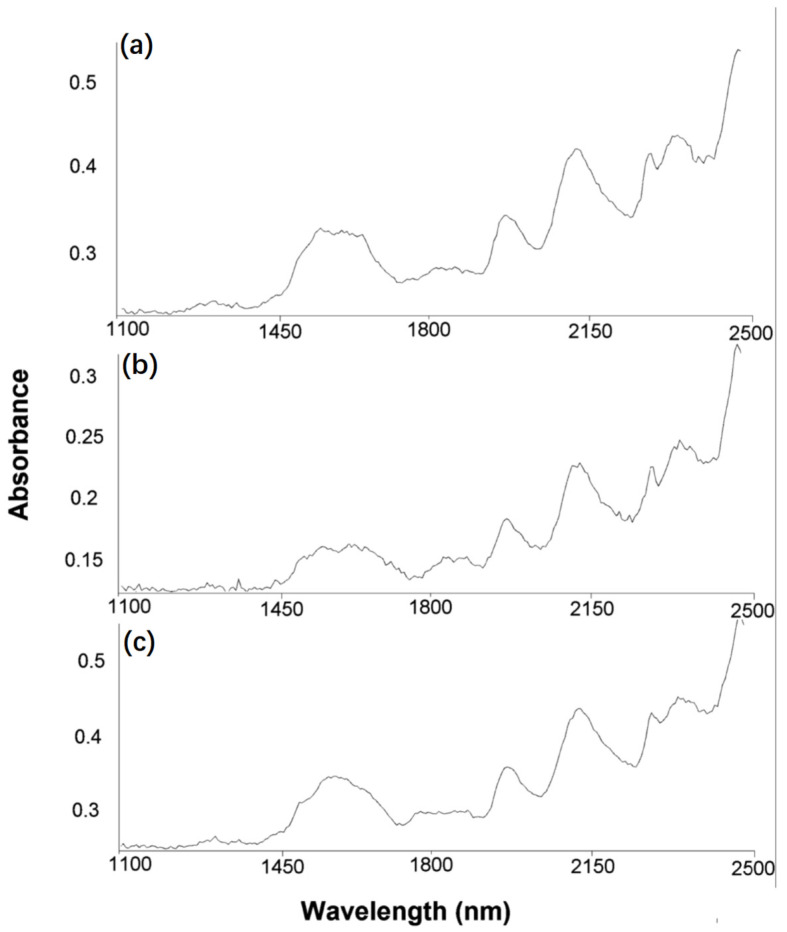
NIR-CI reflectance spectra of RB_204: (**a**) non-dyed cotton fabric; (**b**) dyed cotton fabric, and (**c**) dyed and UV irradiated cotton fabric for 100 h.

NIR absorption bands assigned to the dyed and UV irradiated cotton fabric for 100 h: 1540 nm (O–H stretch first overtone); 1920 nm (C=O stretch second overtone); 2090 nm (C–H combination); 2270 nm (O–H stretch/C–O stretch combination); 2322 nm (C–H stretch/CH_2_ deformation combination); 2500 nm (C–H stretch/C–C and C–O–C stretch) (Figure 16). New hydroxyl groups appear through the cleavage of the ether bond either from dye-cellulose or even from the cellulose structure. Thus, dye entities can leave with monosaccharide moietes from the cotton structure [40]. It was concluded that the UV exposure led to the partial detachment of the dyes from the textile surfaces, together with glucose units and dye photodegradation by destruction of chromophore groups and aromatic rings. NIR spectra showed possible peroxidation processes in the structures of the dyed fabrics with RB_204.

## 4. Conclusions

This paper has explored the range of options available to textile manufacturers to reduce the toxic environmental impact of dyeing cotton textiles with reactive dyes. Structural and color modification of cotton materials dyed with triphenodioxazine structure (Reactive Blue_204) were studied during 100 h UV irradiation time and irradiation dose up to 3500 J cm^−1^. Structural changes during irradiation were compared by applying FTIR, UV-Vis, and NIR-CI techniques. RB_204 is a bis-monofluorothiazine reactive dye characterized by a weaker fixation on the fiber, due to the increased amount of unreacted dye, hydrolyzed from the dye bath under the given conditions. The color intensity increases with increasing amount of dye fixed on the cellulosic fiber. The maximum color intensity occurs at different amounts of dye fixed on the fiber. For Reactive Blue_204, the maximum K/S color intensity was reached for a fiber-fixed amount of dye of 32,181 μg col./g fiber, which corresponds to an initial amount of dye of 120 × 103 μg in the fleet. Depending on the initial mass of the dye, the largest mass losses after irradiation are presented by samples dyed with 1% dye concentration solutions, and the smallest samples dyed with 5% solutions of reactive dyes.

The color changes of the dyed cotton samples, produced after irradiation, were evaluated using colors comparisons and the data were interpreted in the three-dimensional CIELAB system, based on the trichromatic parameters L*, a*, b*.

It was found that ΔE* values (color intensity) increased with increasing irradiation time at all concentrations, the variation resulting in discoloration of irradiated areas, and the highest ΔE* values were obtained for samples dyed with a concentration of 5% dye solution. Another aspect of the research was focused on UV irradiation of dyed samples, followed by their extraction with aqueous solutions in alkaline and acidic pHs, simulating perspiration, compared to non-irradiated samples extracted in neutral medium (distilled water).

The analyzed dye was extracted after irradiation in the largest quantities in the alkaline environment, which would correspond to perspiration with a higher pH, confirming that in the case of an alkaline perspiration, there is a risk that the studied dye will pass through the dyed cotton in the perspiration and thus be able to manifest its harmful effects.

## Figures and Tables

**Table 1:** The characteristic data of RB_204.

Name	Reactive Blue_204 (RB_204)
Structural formula	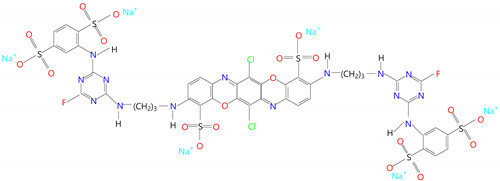
Molecular formula	C_42_H_28_O_20_N_14_S_6_Cl_2_F_2_Na_6_
Molecular weight (M_w_)	1487.97 g/mol
λ_max_	260 nm; 636 nm
IUPAC name	Hexasodium-6,13-dichloro-3,10-bis((4-(2,5-disulfonatoanilino)-6-fluoro-1,3,5- triazin-2-ylamino)prop-3-ylamino)-5,12-dioxa-7,14-diazapentacene-4,11-disulfonate
CAS name	4,11-Triphenodioxazinedisulfonic-acid,6,13-dichloro-3,10-bis((3-((4-((2,5-disulfo- phenyl) amino)-6-fluoro-1,3,5-triazin-2yl)amino) propyl) amino)-, hexasodium salt

**Table 2:** Variation color parameters as a function of irradiation time and radiation dose for RB_204 dyed fabric.

Conc.	Irradiation Time (h)	Radiant Exposure (J cm^−2^ × 10^2^)	L*/(STD)	a*/(STD)	b*/(STD)	ΔE*/(STD)	ΔL*/(STD)	Δa*/(STD)	Δb*/(STD)
1%	0	0	34.5/(0.34)	14.4/(1.64)	−33.2/(1.86)	-	-	-	-
	25	8.8	37.0/(0.75)	12.7/(0.99)	−30.8/(0.53)	3.9/(1.00)	2.5/(0.50)	−1.7/(0.64)	2.4/(0.24)
	50	17.5	37.3/(1.11)	1.1/(1.01)	−27.5/1.21)	7.2/(0.85)	2.8/(0.47)	−3.3/(0.58)	5.7/(0.58)
	75	26.3	35.9/(0.95)	8.3/(0.81)	−24.3/(1.17)	10.9/(0.90)	1.4/(0.43)	−6.1 (0.61)	8.9/(0.79)
	100	35.0	35.1/(1.05)	7.6/(0.78)	−21.0/(1.10)	14.0/(0.98)	0.6/(0.11)	−6.8 (0.50)	12.2 (0.8)
3%	0	0	23.3/(0.85	17.6/(0.51)	−34.2/(0.89)	-	-	-	-
	25	8.8	26.8/(0.76	14.3/(0.68)	−31.2/(0.78)	5.7/(0.82)	3.5/(0.31)	−3.3/(0.31)	3.0/(0.42)
	50	17.5	27.2/(0.68)	13.9/(0.71)	−29.9/(0.86)	6.9/(0.89)	3.9/(0.35)	−3.7/(0.72)	4.3/(0.31)
	75	26.3	24.6/(0.62	13.7/(0.43)	−28.7/(1.21)	7.0/(0.76)	3.0/(0.33)	−3.9/(0.68)	5.5/(0.27)
	100	35.0	23.7/(0.57)	13.0/(0.57)	−25.4/(1.05)	9.9/(0.95)	0.4/(0.1)	−4.6/(0.55)	8.8/(0.50)
5%	0	0	19.7/(0.62)	16.6/(0.44)	−32.9/(0.79)	-	-	-	-
	25	8.9	19.0/(0.51)	14.1/(0.42)	−29.7/(0.87)	4.1/(0.71)	−0.7/(0.1)	−2.5/(0.67)	3.2/(0.6)
	50	17.5	18.4/(0.58)	10.8/(0.54)	−28.7/(0.86	7.3/(0.65)	−1.3 (0.23)	−5.8/(0.63)	4.2/(0.44)
	75	26.3	17.9/(0.65)	8.9/(0.65)	−27.8/(0.95)	9.1/(0.38)	−1.8 (0.21)	−7.7/(0.78)	5.1/(0.30)
	100	35.0	20.2/(0.49)	8.4/(0.71)	−27.1/(0.93)	10.1/(0.24)	0.51 (0.1)	−8.2/(0.28)	5.8/(0.26)

STD = Standard deviation.

**Table 3:** Color intensity and discoloration degree of samples dyed with 1%, 3%, and 5% solution of reactive dye as a function of irradiation time irradiated in UV, measured at 636 nm.

Concentration	Irradiation Time (h)	K/S Values	Discoloration Degree (%)
1%	0	2.24	0
	25	1.71	23.661
	50	1.58	29.464
	100	1.31	41.518
3%	0	4.24	0
	25	3.70	12.736
	50	3.41	19.575
	100	2.89	31.840
5%	0	5.31	0
	25	4.46	16.008
	50	4.39	17.326
	100	3.82	28.060

**Table 4:** The amount of dye existing on the fibers treated with 1%, 3%, and 5% dye concentration solution.

Concentration	Irradiation Time (h)	Dye Mass Variation During Irradiation (μg dye/g Dyed Fiber)
1%	0	5140
	25	3924
	50	3626
	100	3006
3%	0	24,489
	25	21,370
	50	19,695
	100	16,692
5%	0	39,892
	25	33,506
	50	32,980
	100	28,698

**Table 5:** FTIR frequency characteristics of the most important groups and their existing frequencies in the dye molecule RB_204.

Group Type	Group Structure	Frequency	Current FTIR Frequency in the Studied Dye	
			Nonirradiated	Irradiated 100 h
aryl sulfonate	Ar–SO_3_^−^	1230–1120 1080–1025	1062 1087	1064 1086
aryl-chlorine	Ar–Cl	850–700	708	708
fluoro-triazine	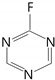	1344		
dioxazine	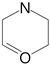	2100	2115	2115
triazine	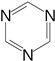	1550 1410	1533	-
alkyl-NH-aryl	–H_2_C–NH–Ar	3450		
propylene	–CH_2_–CH_2_–CH_2_–	740		
etheric	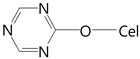	1020–1075 1200–1275	1032 1240	- 1025 1044 1239
(alkyl)_2_NH-	–(CH_2_)_2_N–	3310–3350	3334	3334

**Table 6:** Dependence of absorbents on extraction time and 5% concentration of dye extracted from fiber in a neutral medium.

Number of Determinations	Mass of Sample (g)	Absorbance	Extraction Time (h)	Extracted Dye Concentration (μg/mL)	Amount of Dye Extracted (μg/g Sample)
1	0.1200	0.028	1	0.326	67.916
2	0.1158	0.036	2	0.420	90.674
3	0.1140	0.051	3	0.594	130.263
4	0.1128	0.075	4	0.874	193.706
5	0.1160	0.111	5	1.294	278.879
6	0.1130	0.153	6	1.783	394.469
7	0.1080	0.197	7	2.296	531.481
8	0.1120	0.241	8	2.809	627.009

**Table 7:** Absorbance dependence of irradiation duration and 5% concentration of dye extracted from fiber in an alkaline medium.

Number of Determinations	Mass of Sample (g)	Absorbance	Irradiation Time (h)	Extracted Dye Concentration (μg/mL)	Amount of Dye Extracted (μg/g Sample)
1	0.1043	0.084	1	0.979	234.660
2	0.1050	0.100	2	1.166	277.619
3	0.1055	0.120	3	1.399	331.517
4	0.1047	0.145	4	1.690	403.534
5	0.1060	0.170	5	1.981	467.217
6	0.1061	0.206	6	2.401	565.740
7	0.1040	0.235	7	2.739	658.413
8	0.1053	0.283	8	3.298	783.000

**Table 8:** Dependence of absorbents on irradiation duration and 5% concentration of dye extracted from dyed cotton in an acid medium.

Number of Determinations	Mass of Sample (g)	Absorbance	Irradiation Time (h)	Extracted Dye Concentration (μg/mL)	Amount of Dye Extracted (μg/g Sample)
1	0.1083	0.037	1	0.431	59.695
2	0.1100	0.059	2	0.688	93.818
3	0.1060	0.074	3	0.862	121.981
4	0.1072	0.092	4	1.072	150.000
5	0.1091	0.112	5	1.305	179.423
6	0.1077	0.135	6	1.573	219.081
7	0.1084	0.160	7	1.865	258.072
8	0.1088	0.184	8	2.145	295.726

## Data Availability

Not applicable.
